# Anaerobic Bacteremias in Left Ventricular Assist Devices and Advanced Heart Failure

**DOI:** 10.1155/2019/7571606

**Published:** 2019-12-19

**Authors:** David R. Murillo-Garcia, Julian Galindo, Natalia Pinto, Gabriel Motoa, Esther Benamu, Carlos Franco-Paredes, Daniel B. Chastain, Andrés F. Henao-Martínez

**Affiliations:** ^1^Public Health and Infection Research Group, Faculty of Health Sciences, Universidad Tecnológica de Pereira, Pereira, Colombia; ^2^School of Medicine, Universidad Libre, Cali, Colombia; ^3^Division of Infectious Diseases, University of Colorado Denver, Denver, CO, USA; ^4^Hospital Infantil de México, Federico Gómez, Mexico City, Mexico; ^5^University of Georgia College of Pharmacy, Athens, GA, USA

## Abstract

Left ventricular assisted devices (LVADs) have revolutionized the treatment of advanced heart failure, providing meaningful increases in survival, functional capacity, and quality of life. There are two categories of LVADs patients: (1) bridge-to-transplant and (2) destination therapy. Advanced heart failure and destination LVADs often carry a poor prognosis. The overall 1-year mortality rate remains as high as 30%. LVAD-specific infections, LVAD-related infections, and non-LVAD-related infections represent important emerging clinical problems in this setting. With an incidence ranging from 30 to 50%, these lead to high rates of hospitalization, morbidity, and mortality. Bacteremias caused by anaerobic pathogens in patients with LVAD are underreported. Herein, we describe the microbiological findings, treatment, and clinical outcome of four patients with LVADs and advanced heart failure with anaerobic bacteremias. *Fusobacterium* species was the most frequent etiological agent. Most patients had a relatively favorable short-term outcome with survival rates of 100% at 30 days and of 50% at 90 days. However, due to other multiple long-term complications, overall mortality remains at 50% during the first year and increases to 75% beyond the first year. Anaerobic bacteremia sources included the oral cavity from odontogenic infections and aspiration pneumonia. Anaerobic bacteremia constitutes an unfavorable mortality prognostic factor in patients with destination LVADs. We recommend implementing preventive strategies with a comprehensive dental care evaluation in patients with LVADs and advanced heart failure.

## 1. Introduction

Patients affected with heart failure have improved survival rates thanks to advances in its medical management [1–3]. For individuals who face a high short-term mortality risk and scarce chances of receiving a transplant, left ventricular assist devices (LVAD) have continued to be a therapeutic option [4]. LVADs can provide significant increases in survival, functional capacity, and quality of life [5–7]. Despite these improvements, the overall 1-year mortality rate in LVAD patients remains as high as 30% [8]. There is an increase in the noncardiovascular causes of mortality within this population [9]. Infections represent an important emerging clinical problem leading to decompensation of heart failure (HF) and sepsis [1, 9, 10]. Infections remain a complicating factor of LVAD therapy and HF, with reported rates ranging from 30 to 50% [11, 12]. The importance of controlling infection to optimize survival is of high priority when mechanical circulatory assist devices are used [13]. LVAD-associated infections may be acquired in the community or during hospitalization [10]. The most common infection sites consist primarily of the pulmonary, followed by the urinary tract and skin infections [10, 14]. Reported pathogens involved in cases of LVAD infections are predominantly skin organisms, such as Gram-positive cocci (44.8%) and less often by Gram-negative rods (24.1%) [15]. Among the Gram-positive cocci, the most frequently isolated pathogens are *S. aureus*, coagulase-negative *Staphylococci*, and *Corynebacterium* species; while *P. aeruginosa* and *Enterobacteriaceae* are the most commonly reported Gram-negative rods [16]. Although fungal infections are rare, they can occur and are mainly caused by candida [16, 17]. Anaerobic infections are not typically associated with line or cardiac device infections, and thus, these are often overlooked in patients with LVADs or advanced heart failure. The most common sources of anaerobic bacteremia in the general population are intra-abdominal abscesses and female genital tract infections. Data on their clinical presentation, severity, diagnosis, treatment, and prognosis in this setting are lacking. In the present case series, we perform a description of the microbiological findings, treatment, and clinical outcome of four anaerobic pathogen infections in patients with LVADs and advanced heart failure.

## 2. Case Series

### 2.1. Patient 1

A 28-year-old man with a past medical history of nonischemic cardiomyopathy with an ejection fraction of 10% with a HeartMate II LVAD placement since eight months, pulmonary hypertension due to chronic thromboembolic disease, obstructive sleep apnea, obesity, and substance abuse presented to the emergency department with fever (102 F°) and cough that started two days before. He was hemodynamically stable. His SpO2 was 95%. On physical examination, he had decreased breath sounds and rhonchi in the right lower field. There were no abdominal symptoms or physical exam findings concerning for intestinal perforation, and no history of intravenous drug abuse.

A chest x-ray showed a consolidative airspace opacity involving the right lobe and no pleural effusion. He was initially treated with piperacillin-tazobactam. Transthoracic echocardiogram (TTE) revealed stable findings. Blood cultures grew anaerobic Gram-negative rods speciated as *Fusobacterium nucleatum*. Driveline infection was not suspected, and cultures were not obtained. His antimicrobial therapy was switched to metronidazole. Computed tomography (CT) with contrast did not show evidence of LVAD-related abscess or LVAD cannula thrombus. Antimicrobial susceptibilities confirmed that the organism was sensitive to metronidazole, and the patient was discharged on day 4 of hospitalization in stable condition to complete a 14-day course. Surveillance cultures were negative, and control chest x-ray evidenced improvement of the previous right lower lobe consolidation. The source of his anaerobic bacteremia was his lower respiratory tract, which might be due to aspiration in the setting of obstructive sleep apnea, substance abuse, and obesity.

Three years later, the patient was diagnosed with necrotizing pneumonia. He rapidly developed multiorganic failure that was presumably due to an additional underlying infection of the LVAD. He was deemed a poor candidate for a heart transplant due to poor compliance with prior treatments. His family agreed for his transition to comfort care measures.

### 2.2. Patient 2

A 78-year-old Caucasian male with a past medical history of type 2 diabetes, hypertension, atrial fibrillation, gout, and ischemic cardiomyopathy with chronic severe systolic heart failure (ejection fraction of 30%) underwent a HeartMate XVE LVAD placement. His course was complicated by pump failure requiring LVAD exchange with a HeartMate II 2 years later and multiple episodes of driveline site infections due to *Klebsiella pneumoniae*. He presented with a recurrent episode of gastrointestinal (GI) bleeding, INR lability, and positive blood cultures for *Bacteroides thetaiotaomicron*. New driveline infection was not suspected then. He received aztreonam and metronidazole empirically. Chest CT showed a moderate left pleural effusion with atelectasis of the left lower lobe. The clinical assessment identified two potential sources for the bacteremia, including pneumonia with a parapneumonic effusion and bacterial translocation in the setting of GI bleeding. He was discharged home with readmission the same day due to GI bleeding and new positive blood cultures for *Citrobacter freundii*. He was started on ceftazidime and vancomycin. Repeated blood cultures showed clearance. The patient was sent home on trimethoprim/sulfamethoxazole, metronidazole, and rifampin for chronic suppression. The trimethoprim/sulfamethoxazole and rifampin were chosen to cover *Enterobacteriaceae* with increased biofilm penetration with rifampin. Metronidazole was covering the *Bacteroides thetaiotaomicron*. Seven months later, he was readmitted with acute GI bleeding, candidemia, and recurrent *Citrobacter freundii* bacteremia refractory to therapy. The patient continued to decline clinically, and he was eventually transitioned to comfort care.

### 2.3. Patient 3

A 47-year-old man with a past medical history of nonischemic cardiomyopathy with an ejection fraction of 10–15% and prior history of ethanol abuse presented to the emergency department with seizures and cardiogenic shock with features of acute renal failure, coagulopathy, shock liver, and pulmonary edema and required venoarterial extracorporeal membrane oxygenation. As he was not a candidate for heart transplantation, an HeartMate II LVAD was placed. As part of the workup at our institution, the patient had surveillance blood cultures before LVAD placement. Five days later, 1 out of 2 blood culture bottles became positive for *Fusobacterium nucleatum*. The patient was initially treated with piperacillin/tazobactam, and then switched to metronidazole two days later when the susceptibilities became available (resistant to piperacillin). We did not have initial concerns for a LVAD infection, and cultures were not obtained. CXR showed extensive airspace opacities, right greater than left. The source was presumed to be GI transient translocation vs. aspiration pneumonia. After completing 14 days of treatment, blood cultures were cleared, and a TTE showed no vegetations. The patient was discharged home. Two weeks later, he was readmitted with an elevated lactate dehydrogenase (LDH) and had urgent LVAD replacement for presumed LVAD thrombosis. Intraoperative cultures were positive for *Rhizopus spp.*, and a CT scan showed possible postsurgical changes vs. an abscess around the area of the LVAD. Possible source of the mold was likely an undetectable fungemia with lungs as the most common port of entry. Universal fungal prophylaxis is not usually performed in this setting. The patient was started on indefinite therapy with posaconazole and received six weeks of vancomycin. A week later, the patient was readmitted with cardiogenic shock, lactic acidosis, and hypoglycemia. The patient developed LVAD thrombosis. He then developed a series of hemorrhagic complications, including epistaxis and subdural hematoma, leading to pulseless electrical activity arrest and LVAD compromise. The family declined more aggressive management, and the patient was transitioned to comfort care.

### 2.4. Patient 4

A 65-year-old man with severe ischemic cardiomyopathy status after placement of an LVAD, type 2 diabetes, tobacco dependence, and chronic methicillin-sensitive *Staphylococcus aureus* (MSSA) LVAD infection on chronic suppressive therapy with cephalexin presented with acute onset of fever and driveline site discharge. Blood cultures grew *Parvimonas micra*, with repeat positive culture six days later, suspicious for LVAD infection. Driveline site culture grew *Corynebacterium spp.* of unclear significance since this bacteria is a common skin colonizer. He had poor dentition on physical exam. CXR revealed a small/moderate right pleural effusion with mild right basilar atelectasis. TTE showed new multiple small echodensities visible in the short axis view of the aorta of unclear etiology. The primary source was thought to be his poor dentition complicated with a presumed LVAD infection. He received a six-week course of therapy with PO metronidazole with plans of transition to amoxicillin-clavulanate or doxycycline for chronic suppression of his previous infections. Thirty days after his initial bacteremia, he was still alive but readmitted with rising LDH and hematuria concerning for LVAD thrombosis, with no signs of recurrent infection.

## 3. Discussion

To the best of our knowledge, this report is the first to describe the clinical features of anaerobic bacteremia in patients with LVADs and severe heart failure. At admission, half of patients presented with septic shock. We found *Fusobacterium* species as the most frequent etiological agent (2 out of 4 cases). The most common identifiable source was of pulmonary origin in the setting of aspiration pneumonitis/pneumonia. Other possible relevant sources included dental cavities and transient GI translocation (Table 1). Driveline and LVAD-related infections were commonly present either at presentation or as a complication. All patients were initially treated with a broad-spectrum penicillin or cephalosporin and subsequently de-escalated to metronidazole. Three patients had proven recurrent bacteremia with other organisms. Chronic suppressive antibiotics were used in LVAD-related infections. Most patients had a relatively favorable short-term outcome with survival rates of 100% at 30 days and of 50% at 90 days. However, due to other multiple long-term complications, 1-year mortality stayed at 50% and increased to 75% beyond the first year following the initial episode of anaerobic bacteremia. Causes of death included other infectious etiologies highlighting the high mortality prognostic factor of the anaerobic bacteremia. The 30-day and 1-year mortality rates correlate with findings previously described in the literature [1, 14, 18]. Most patients had destination LVADs, a reflection of the patients' comorbidities and inability to undergo heart transplant. We hypothesize that the proportion of anaerobic bacteremia of all bacterial infections among LVADs recipients is a minority. This case series is the first clinical description of this type of infection for this patient population.

Advanced forms of heart failure have a poor prognosis, which is aggravated by the presence of comorbidities [18]. Improvements in the treatment of cardiovascular disease have been associated with a progressive increase in the prevalence of heart failure, which has led to the rise in deaths due to noncardiovascular pathologies in LVAD recipients [10, 18]. In this context, infections represent a significant emerging clinical problem, with increased mortality rates [10, 19].

LVAD infections are a common cause of morbidity with an estimated 1-year mortality rate of 20%. Moreover, in patients who develop an infection, mortality is 5.6 times higher [12, 20, 21]. Infections are also one of the leading causes of readmission in these patients [22]. LVAD-related infections are also associated with an increased risk for pump thrombosis, stroke, bleeding, prolonged hospitalizations, need for LVAD exchange, and failure to transplant.

Pneumonia and sepsis are the most common infectious complications present in patients with advanced heart failure and LVAD recipients [1, 10, 12, 18]. Driveline site infections follow in third place, occurring in approximately 19% of LVAD recipients within one year after implantation [12]. Management of bacteremia in this setting is challenging. Without an identifiable source, LVAD infections should always be suspected. Clinical assessment should pursue a driveline exit site examination looking for evidence of erythema, warmth, and purulent drainage and workup consistent with blood cultures, chest radiography, and transesophageal echocardiography. As we described in our case series, pneumonia, aspiration pneumonitis, and associated dental infections are the most common sources of anaerobic bacteremia and a significant cause of morbidity in patients with LVADs and advanced heart failure. Transient GI translocation due to the low output-associated mucosal ischemia may be a less common source to consider. Continuous-Flow LVAD support causes a distinct form of intestinal angiodysplasia which presumably is responsible for GI bleeding and translocation of intestinal bacteria [4, 23]. LVADs infection can also predispose or complicate any bacteremia including those caused by anaerobes. However, the limited number of patients in our series precludes any establishment of strong causal associations.

Bacteremia treatment will always depend on the source of the infection. In LVAD nonrelated bacteremias, clinicians should direct the courses and type of antibiotics based on the main source of infection (pneumonia, skin and soft tissue infections, etc.). Management of LVAD-related bacteremias and infections is complex and depend on several factors: type of LVAD and heart transplant candidacy, infection extension, endocarditis, mediastinitis, or isolated driveline infections, and presence of metastatic foci, type of bacteria, and complications. They usually require 6-week courses followed by chronic suppressive antibiotics. Suppressive antibiotics are given to decrease the risk of relapses in LVAD-related infections since bacteria can persist in the devices' biofilms.

Infection and sepsis contribute significantly to mortality in LVAD recipients [1, 10, 12, 20, 21]. Our results suggest that anaerobic bacteremia can be considered as an adverse prognostic factor of mortality in patients with destination LVADs. Infections with anaerobic bacteria originating from the oropharyngeal flora can complicate with bacteremia or aspiration pneumonia. Thus, we recommend implementing preventive strategies with a comprehensive dental care evaluation in patients with LVADs and advanced heart failure.

## Figures and Tables

**Table 1:** Relevant clinical features of patients with anerobic bacteremias.

Clinical feature	Patient 1	Patient 2	Patient 3	Patient 4
Sex	Male	Male	Male	Male
Age	28	78	47	65
Comorbidities	pHTN, OSA, IDA, obesity	Dilated cardiomyopathy, HTN, Afib, T2DM, gout	Nonischemic cardiomyopathy	T2DM, current smoking
LVAD present	Yes	Yes	Yes	Yes
Driveline cultures	No	Yes (previously)	Yes (during second admission)	Yes
LVAD-related infection	No	Yes (history of)	Yes (as a complication)	Yes
Risk factors	Drug abuse, former smoker	Liable INR, several GI bleeding	Anticoagulation	Poor dentition LVAD infection
Isolated bacteria	*Fusobacterium nucleatum*	*Bacteroides thetaiotaomicron/Citrobacter Freundii*	*Fusobacterium nucleatum*	*Parvimonas micra*
Source	Lung (PNA)	PNA vs. transient GI translocation	PNA	Mouth
Outcome	Improvement	Severe septic shock	Cardiogenic shock and coagulopathy	LVAD thrombosis
Treatment	MTZ	MTZ	MTZ	MTZ
Recurrent bacteremia	No	Yes	Yes	Yes
GI bleeding	No	Yes	No	No
Mortality				
30 days	No	No	No	No
90 days	No	No	Yes	No
1 year	No	Yes	Yes	N/A
Deceased	Yes	Yes	Yes	No
Cause of death	Septic shock due to *S. aureus* bacteremia	Septic shock due to *Citrobacter freundii*	LVAD thrombosis, *Rhizopus* pocket infection, and hemorrhagic stroke	N/A

Afib: atrial fibrillation, GI: gastrointestinal, HTN: hypertension, IDA: iron deficiency anemia, LVAD: left ventricular assisted device; MTZ: metronidazole, OSA: obstructive sleep apnea, pHTN: pulmonary hypertension, PNA: pneumonia, T2DM: type 2 diabetes mellitus.
